# Supplementary figures and images for: Longitudinal study of leukocyte DNA methylation and biomarkers for cancer risk in older adults
Source: Biomark Res. 2019 May 28;7:10. doi: 10.1186/s40364-019-0161-3 (PMC6537435; doi:10.1186/s40364-019-0161-3)

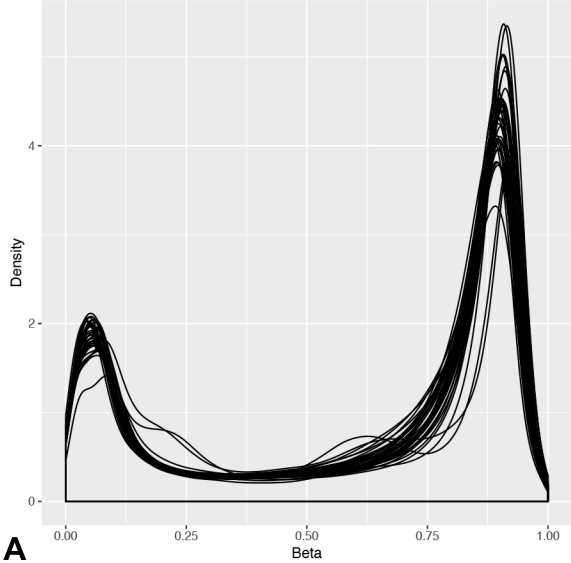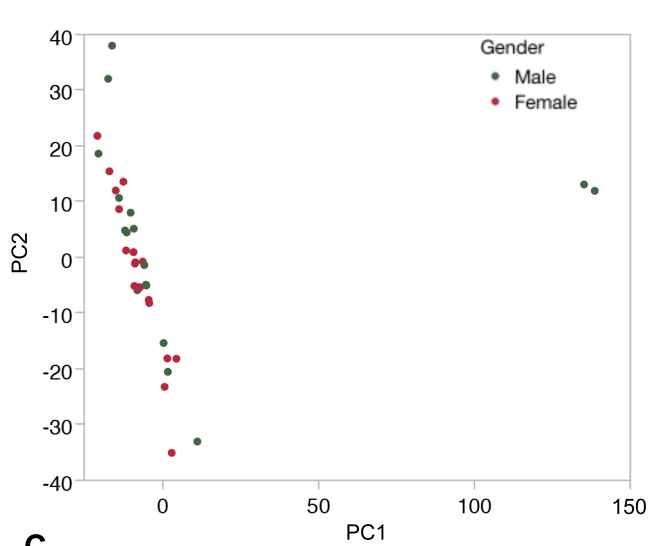

**A**

**C**

Height

**B**

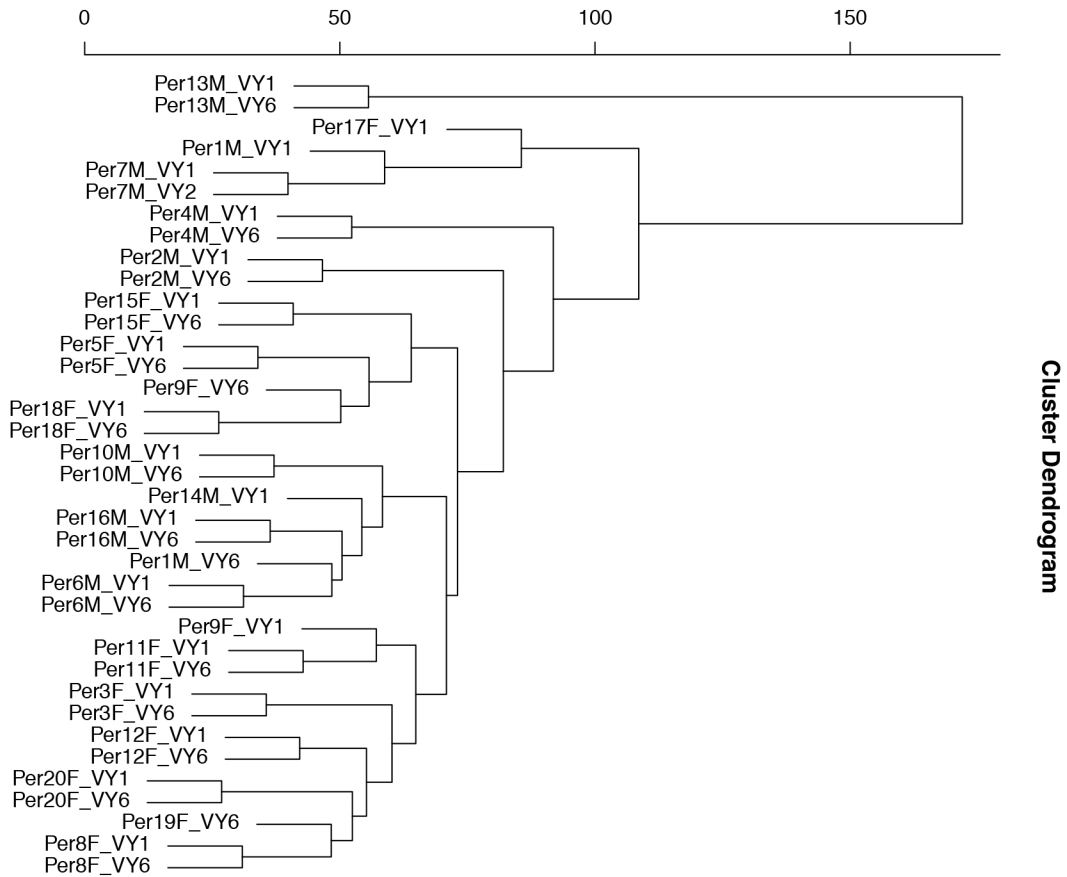

Supplement: Supplementary file 1 — Figure S1. Microarray data quality checks. (A) The density plots for β-values using the full set of 866,836 probes show the expected bimodal distribution. (B) Unsupervised hierarchical clustering using the full set of probes shows that, with the exception of two participants (Per1 and Per9), all samples with longitudinal data pair appropriately with self. This cluster tree identifies Per13 as an outlier at both baseline and visit year 6. (C) Principal component analysis was done using a filtered set of 739,648 autosomal probes. The scatter plot between principal component 1 (PC1) and PC2 identifies Per13 as an outlier. (PDF 1310 kb) [file 40364_2019_161_MOESM1_ESM.pdf]

Height

5 10 15 20 25 30

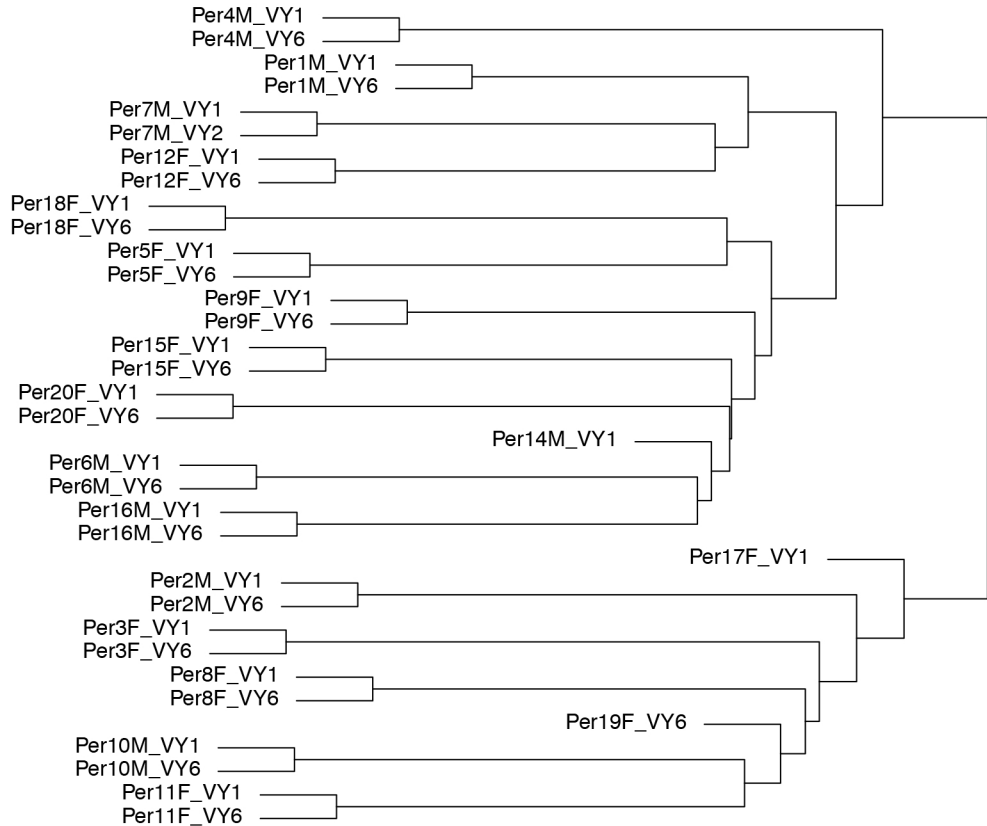

Cluster Dendrogram

Supplement: Supplementary file 2 — Figure S2. Samples pair by participant ID. Unsupervised hierarchical clustering using probes that were flagged due to overlap with SNPs shows that samples collected longitudinally from the same participant pair perfectly. (PDF 495 kb) [file 40364_2019_161_MOESM2_ESM.pdf]
